# Supplementary material for: Sustained-input switches for transcription factors and microRNAs are central building blocks of eukaryotic gene circuits
Source: Genome Biol. 2013 Aug 23;14(8):R85. doi: 10.1186/gb-2013-14-8-r85 (PMC4054853; doi:10.1186/gb-2013-14-8-r85)
Supplement: Additional file 5 — HTML Browsable Motif Output. Zipped folder containing all WaRSwap and FANMOD motif output, viewable in a web browser. [file gb-2013-14-8-r85-S5.ZIP › HTML_browsable_motif_output/WaRSwap_ath_TwoLoops_withSelfLoops/sigs_WSWRSLDraw_TAIR10_TwoLoops_SLColors-2500.pvals.heatmaps.html/motif_id_6_3113_tftype_ath_upstream_-3000_0.html]

```
BG_MODEL = WARSwap
MOTIF_ID = 6_3113
TF_TYPE = ath
UPSTREAM = -3000_0


PVals
FNR = 0.2	FNR = 0.4	FNR = 0.6	FNR = 0.8
deltaG = 60	0.002	0.047	0.004	0.172
deltaG = 70	0.005	0.038	0.01	0.152
deltaG = 80	0.004	0.046	0.006	0.152

ZScores
FNR = 0.2	FNR = 0.4	FNR = 0.6	FNR = 0.8
deltaG = 60	2.573	1.758	3.293	-0.408
deltaG = 70	2.527	1.82	3.129	-0.373
deltaG = 80	2.534	1.727	3.263	-0.382

StDevs
FNR = 0.2	FNR = 0.4	FNR = 0.6	FNR = 0.8
deltaG = 60	17.295	10.747	3.035	0.514
deltaG = 70	17.827	10.385	3.152	0.517
deltaG = 80	17.737	10.717	3.052	0.491
```
